# Bridging the Gap in Quality Among High Medicaid Nursing Homes: The Role of Management and Community Factors

**DOI:** 10.1093/geroni/igaa057.2361

**Published:** 2020-12-16

**Authors:** Robert Weech-Maldonado

## Abstract

Nursing home quality has been a matter of long-standing policy interest at the federal and state level, as it concerns the health and well-being of one of our most vulnerable populations. Mor et al. (2004) described the nursing home industry as a ‘two-tiered’ system, with the lower-tier nursing homes operating in a resource-constrained environment given their high proportion of Medicaid residents (85% or higher). Medicaid is the largest payer of nursing homes but its reimbursement rates typically lag Medicare as well as private pay. Lower tier facilities are characterized by lower professional staffing and occupancy rates, and worse quality. Such facilities have a higher proportion of minority residents and are generally located in communities with significant proportions of poor and minority residents, exacerbating the existing disparities in nursing home care. However, there are performance variations among high Medicaid nursing homes, with some facilities performing significantly better than others on both quality and financial performance. What may explain the superior performance of certain nursing homes that are operating in a similarly resource-constrained environment? Factors, such as management resources and environmental resource availability, may be the critically important differentiators. The purpose of this symposium is to examine the organizational/management and community factors that may be associated with high-performance among a similar group of resource-constrained nursing homes. Using survey, secondary, and qualitative data analysis, this symposium will explore the role that culture change, leadership style, human resource management practices, knowledge management, and community factors can have on nursing home performance.

